# Study on Volumetric, Compressibility and Viscometric Behavior of Cationic Surfactants (CTAB and DTAB) in Aqueous Glycyl Dipeptide: A Thermo-Acoustic Approach

**DOI:** 10.3390/molecules27248767

**Published:** 2022-12-10

**Authors:** Santosh Kumari, Suvarcha Chauhan, Kuldeep Singh, Ahmad Umar, Hassan Fouad, Mohammad Shaheer Akhtar

**Affiliations:** 1 Department of Chemistry, Himachal Pradesh University, Summer Hill, Shimla 171005, India; 2 Department of Chemistry, MCM DAV College, Kangra 176001, India; 3 Department of Chemistry, College of Science and Arts, and Promising Centre for Sensors and Electronic Devices (PCSED), Najran University, Najran 11001, Saudi Arabia; 4 Department of Materials Science and Engineering, The Ohio State University, Columbus, OH 43210, USA; 5 Applied Medical Science Department, Community College, King Saud University, P.O. Box 10219, Riyadh 11433, Saudi Arabia; 6 School of Semiconductor and Chemical Engineering, Jeonbuk National University, Jeonju 54896, Republic of Korea; 7 Graduate School of Integrated Energy-AI, Jeonbuk National University, Jeonju 54896, Republic of Korea

**Keywords:** glycyl dipeptide, volumetric, apparent molar volume, isentropic compressibility, dehydration

## Abstract

This study aims to understand how glycyl dipeptide affected the compressibility, volumetric behavior and viscometric behavior of the cationic surfactants CTAB (Cetyltrimethylammonium bromide) and DTAB (dodecyltrimethylammonium bromide). Information on solute–solute, solute–solvent, and solvent–solvent interactions has been inferred using the quantification of density (*ρ*), speed of sound (*u*) and viscosity in aqueous media containing glycyl dipeptide in the temperature range 293.15–313.15 K at an interval of 5 K. The data from the aforementioned research have been used to enumerate numerous volumetric and compressibility metrics that aid in the collection of information about the interactional behavior of the system under consideration. The study suggests that CTAB interacts strongly compared to DTAB with dipeptide, and it also significantly dehydrates glycyl dipeptide. The difference in water–water interactions caused by the loss of hydrophobic hydration of the surfactant molecules upon the addition of cationic surfactants may be the cause of the variation in determined parameters with surfactant concentration. Consideration of the structural rearrangement of molecules that may occur in the system has been used to explain the results of viscosity and computed factors related to viscosity. The patterns of competitive intermolecular interactions in the ternary (dipeptide + water + surfactant) system have been used to analyze the trends of all the parameters. The study may be helpful to understand the stability and structural changes in protein–surfactant systems mediated through various interactions that may be present in the system.

## 1. Introduction

Micellar characteristics of amphiphiles such as surfactants are appreciably impacted by the aqueous environment containing peptides [1]. Small peptides and amino acids are referred to as water structure influencing molecules, having a noteworthy effect on the aqueous system. The systematic study of peptide–surfactant interactions can provide valuable information about their behavior in solution and continues to be an imperative and mounting area of exploration. In the recent years, there have been various methods and deliberate interactions that havebeen proposed and widely known to study the relationship between surface active agents and bio-active molecules in aqueous solution. The two chemical elements that best describe living things are peptides and proteins. Due to their size, dietary proteins are disproportionately smaller compounds involving dipeptides and amino acids, and are absorbed via different routes. When compared to amino acids, dipeptides are absorbed more quickly [2]. Peptides have an important role in biological and physiological processes and are essential to many areas of biomedical research [3,4]. Given that peptides are the primary building blocks of many complex biomolecules, including proteins and enzymes, the improved performance of dipeptides has piqued our interest in investigating the current work. They are also significant biomolecules because they have a wide range of uses in the production of drugs due to their capacity to function as transmitters and hormones [5].

Peptides being significant molecules have their remarkable applications in drug production and signal transmission in cell communication [5,6]. The methodical study of peptides provides precious information and insight for acute observation into the conformational stability of proteins and their behavior in solutions. In some earlier reported thermodynamic analyses, the strong influence on hydration of additives like inorganic salts, surface active agents or bio-imperative compounds in aqueous rich peptide solution has been advocated [7,8,9]. Such behavior results in significant adjustments to their capacity to bind to other molecules. Because they alter the way that proteins operate, peptide–surfactant interactions are crucial. Therefore, understanding the nature of these interactions is crucial. Since nearly all biochemical activities occur in aqueous media, research on the physicochemical properties of biomolecules such as amino acids, peptides, proteins and sugars in aqueous solution is useful in understanding the intricate mechanism of molecular interactions [9]. Numerous practical domains, including physiology, computer-aided design and the optimization of chemical processes in businesses, require the derived thermodynamic data. Additionally, rather than using amino acids, tiny peptides have been favored, since they more closely resemble proteins due to their complexity [10].

Surfactants, on the other hand, are the fundamental components of colloid chemistry because they are schizophrenic molecules with distinct polar and non-polar domains. Because of their significance in technology and scholarly interest, they identify as a subset of colloid chemistry. The in-depth research on surfactants [11,12,13,14,15,16,17,18,19] gave colloidal chemistry a stronger foundation. Self-aggregation of surfactants in solutions, which enables their use in emulsion stabilization, detergency, drug delivery targeting, etc., is their most crucial attribute. They have many uses and applications in different fields of research and technology, especially in pharmaceutical and biotechnological process, and their properties are studied with a great curiosity to enrich the literature for information. In addition, surfactants, including collectors, are also widely used in the field of mineral flotation to improve the recovery of minerals [20,21]. There have been some investigations on the volumetric, compressibility and viscometric properties, and fewer studies on interactions of CTAB and DTAB in aqueous solution containing glycyl dipeptide [22].

**Figure 1:**
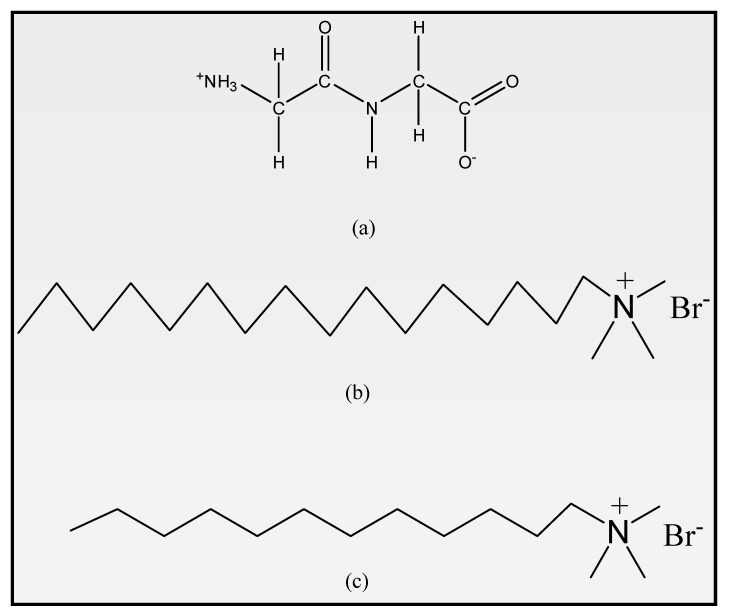
Schematic representation for chemical structure of (**a**) Glycyl dipeptide, (**b**) CTAB and (**c**) DTAB.

We attempt to measure some physico-chemical properties of cationic surfactants CTAB and DTAB in an aqueous solution containing glycyl dipeptide using various techniques, such as density, speed of sound and viscosity analysis, in light of the aforementioned information and our curiosity in investigating the interaction of dipeptide and surfactant. This will reveal the various intermolecular interactions between CTAB/DTAB and dipeptides. Volumetric and viscometric characteristics, including apparent molar volume, apparent molar adiabatic compression, apparent molar isentropic compressibility, and viscous relaxation time, offer important insights into the interactions between solutes and solvents in the solution phase. The study may be ready to lend a hand on extracting information regarding the power of surfactants to extend the protein conformational changes of thenative and denatured state of proteins. The chemical structure of glycyl dipeptide, CTAB and DTAB have been presented in Figure 1a–c:

## 2. Materials and Methods 

### 2.1. Material

The Millipore-Elix equipment was used to makethe deionized water for the study. Deionized water with a conductivity of (2 to 3) ×10^−6^S∙cm^−1^ and pH of 6.8 to7.0 at a temperature equal to 298.15 K was made. Himedia Pvt. Ltd. (Mumbai, India) has provided CTAB and DTAB of AR grade. The well-known procedure that is described elsewhere [23] was used to recrystallize the surfactants (CTAB and DTAB) in ethanol. The glycyl dipeptide has been used as-is, without any extra treatment, and it was obtained from Spectrochem Pvt. Ltd. (New Delhi, India). Pyrene (A.R. grade) was purchased from Merck (Darmstadt, Germany)and employed as a fluorescent probe. Table 1 also contains descriptions of each chemical that was used in this study.

### 2.2. Methods

Using an Anton Paar DSA-5000 equipment, the density and sound speed values of surfactant solutions in the presence and absence of glycyldipeptide were simultaneously examined. The operating system and calibration process of the used instrument is already reported in our previous publication [24]. The uncertainty in density and speed of sound measurements is ±2 × 10^−6^g∙cm^−3^ and ±0.2m∙s^−1^, respectively.

The Man Singh Survismeter has been used to measure the efflux time, which is to be incorporated for the calculation of viscosity. The working procedure and calibration method of the Man Singh Survismeter which was purchased from Spectro Lab. Equip. Pvt. Ltd. (New Delhi, India), has already been described in earlier reports [25,26]. Since the efflux time for viscosity measurements was between 300 and 395 s [27], no kinetic energy correction was performed. The Survismeter’s temperature was held constant to within ±0.1 K. For calibration of Survismeter, dimethylsulfoxide and methanol, both of A.R. grade, have been used. The coefficient viscosities of dimethylsulfoxide and methanol were found to be 2.00 and 0.55 centipoise, respectively, which were in resemblance with earlier reports [28].

## 3. Result and Discussion

### 3.1. Volumetric and Compressibility Studies

**Figure 2:**
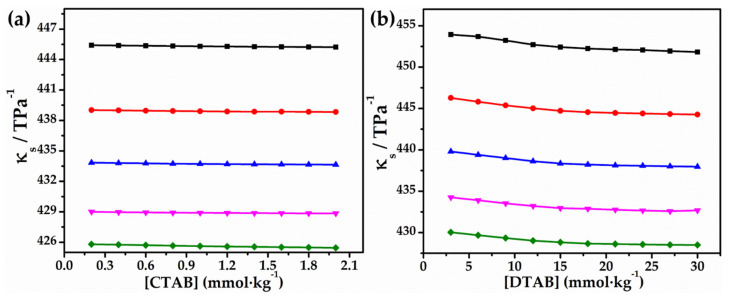
Plots of $\kappa_{s}$ versus (**a**) [CTAB] and (**b**) [DTAB] in 0.010 mol∙kg^−1^ aqueous solution of glycyl dipeptide 293.15 K (■), 298.15 K (●), 303.15 K (▲), 308.15 K (▼) and 313.15 K (◆).

For cationic surfactants CTAB and DTAB in pure water and aqueous solution of glycyl dipeptide, density and sound speed values have been evaluated at various concentrations. The findings are based on the numerous surfactant–dipeptide interactions in aqueous medium that can be inferred from the apparent molar volumes $\left(V_{\phi }\right)$ and apparent molar isentropic compression $\left(\kappa_{S,\phi }\right)$ of both surfactants in glycyl dipeptide solutions. Tables S1 and S2 that are provided in the supporting information provide the experimental density and sound speed values. The quantitative information regarding density and speed of sound have been meticulously used for determination of compressibility parameters, viz., apparent molar volume $\left(V_{\phi }\right)$ and apparent molar isentropic compression $\left(\kappa_{S,\phi }\right)$, by using the following relations [29,30,31]:

(1) $$V_{\varphi }=\frac{M}{\rho }+\frac{\left[\rho_{o}-\rho \right]}{m\rho \rho_{o}}$$

(2) $$\kappa_{s,\varphi }=V_{\varphi }\kappa_{s}+\frac{\left(\kappa_{s}-\kappa_{o}\right)}{m\rho_{o}}$$

where m is the molality of the solution determined as [31]:

m=1/[d/C−M/1000]

here *C* signifies molar concentration and *M* for relative molar mass of surfactant, *ρ* and *ρ_o_* are the densities of the pure solvent and solution, $\kappa_{s}$ and $\kappa_{o}$ are the isentropic compressibilities of the solvent and solution, respectively, and have been determined as: $\kappa_{s}=1/u^{2}\rho$ and $\kappa_{o}=1/{u_{o}}^{2}\rho_{o}$ [32,33]. The magnitudes of the isentropic compressibility, $\kappa_{s}$ for aqueous solutions of CTAB and DTAB, have been given in Table 2 and graphically represented in Figure 2. It is worth mentioning here that the data obtained for apparent molar volume varies in a non-linear fashion and hence could not be analyzed in terms of Masson’s equation of the type [32],

Vφ=Vφo+SV*m12

The $\left(V_{\phi }\right)$ and $\left(\kappa_{S,\phi }\right)$ values calculated from Equations (1) and (2) for aqueous solution of CTAB and DTAB have been tabulated in Table 3 and Table 4. However, the solution behavior of such surfactant, CTAB and DTAB, in the present case due to their amphiphilic nature, i.e., presence of both hydrophobic and hydrophilic groups, they show a mild balance of hydrophobic and hydrophilic interactions [33,34,35]. As apparent molar volume $\left(V_{\phi }\right)$ and apparent molar isentropic compression $\left(\kappa_{S,\phi }\right)$ are quite sensitive to specific and non-specific interactions involving solute–solute and solute–solvent interactions, enumeration of these parameters forms an understanding in respect to the solution behavior of CTAB and DTAB. Thus, it would be very fascinating to analyze the behavior of these parameters in aqueous solutions of the glycyldipeptide under varying experimental conditions (temperature and glycyl dipeptide concentration). Specifically, the following types of interactions may play a role in the ternary system of glycyl dipeptide and cationic surfactants in aqueous medium [36]:

(a) interactions between the N^+^CH3group of DTAB/CTAB and the COO^−^group of glycyl dipeptide or between the Br^−^ of CTAB/DTAB and the NH^3+^ group of glycyl dipeptide,

(b) interactions between the hydrophilic portion of the glycyl dipeptide and the ionic head group of CTAB/DTAB,

(c) contacts between the hydrophobic and hydrophilic groups of the glycyl dipeptide and the alkyl chains of the cationic surfactants, respectively, and (d) interactions between the hydrophobic tail and hydrophobic side groups of the cationic surfactant and the glycyl dipeptide, respectively.

**Table 3:** Apparent molar volume, $V_{\varphi }$/10^−4^ (m^3^∙mol^−1^) values for CTAB and DTAB in pure water and 0.001, 0.005 and 0.010 mol∙kg^−1^ aqueous solution of glycyl dipeptide at different temperatures and pressure *p* = 100 kPa.

CTAB						DTAB					
[CTAB] mmol∙kg^−1^	293.15 K	298.15 K	303.15 K	308.15 K	313.15 K	[DTAB] mmol∙kg^−1^	293.15 K	298.15 K	303.15 K	308.15 K	313.15 K
**[Pure Water]**											
**0.2**	−477.80	−197.71	−617.39	265.41	−343.65	**3**	2.80	2.81	2.84	2.89	2.90
**0.4**	−61.39	76.34	−135.79	298.29	−74.52	**6**	2.84	2.86	2.88	2.91	2.93
**0.6**	74.06	159.31	19.70	310.93	65.97	**9**	2.86	2.87	2.89	2.92	2.93
**0.8**	138.03	204.56	99.97	319.79	134.94	**12**	2.87	2.88	2.90	2.92	2.94
**1.0**	178.41	231.71	150.14	327.12	176.32	**15**	2.88	2.88	2.90	2.92	2.94
**1.2**	208.68	252.33	185.28	331.17	206.45	**18**	2.88	2.89	2.91	2.92	2.95
**1.4**	229.58	267.05	206.77	335.50	225.79	**21**	2.89	2.89	2.91	2.93	2.95
**1.6**	244.63	276.21	223.52	338.12	242.21	**24**	2.89	2.90	2.91	2.93	2.95
**1.8**	254.11	285.57	237.10	337.91	254.41	**27**	2.89	2.90	2.92	2.93	2.95
**2.0**	262.19	292.55	245.95	337.74	264.16	**30**	2.89	2.90	2.92	2.94	2.95
**[Glycyl Dipeptide] = 0.001 mol∙kg^−1^**											
**0.20**	−73.04	−62.09	−52.90	−49.19	−33.12	**3**	0.97	1.25	1.68	2.09	2.40
**0.40**	−35.10	−29.67	−25.20	−23.29	−15.15	**6**	2.02	2.16	2.42	2.66	2.72
**0.60**	−22.58	−18.81	−15.92	−14.58	−9.15	**9**	2.36	2.58	2.70	2.76	2.81
**0.80**	−16.26	−13.34	−11.22	−10.27	−6.20	**12**	2.54	2.69	2.70	2.79	2.84
**0.10**	−12.45	−10.11	−8.45	−7.62	−4.45	**15**	2.60	2.74	2.75	2.80	2.86
**1.20**	−9.92	−7.93	−6.57	−5.84	−3.35	**18**	2.66	2.77	2.78	2.83	2.88
**1.40**	−8.10	−6.37	−5.23	−4.61	−2.52	**21**	2.64	2.78	2.80	2.86	2.89
**1.60**	−6.74	−5.19	−4.20	−3.68	−1.85	**24**	2.68	2.77	2.82	2.86	2.90
**1.80**	−5.69	−4.26	−3.40	−2.96	−1.33	**27**	2.67	2.80	2.83	2.87	2.91
**2.00**	−4.85	−3.51	−2.77	−2.35	−0.88	**30**	2.70	2.82	2.84	2.89	2.92
**[Glycyl Dipeptide] = 0.005 mol∙kg^−1^**											
**0.20**	−67.33	−42.27	−32.28	−21.86	−11.96	**3**	1.28	1.49	1.95	2.40	2.56
**0.40**	−32.12	−19.96	−14.79	−9.55	−4.40	**6**	2.16	2.27	2.51	2.72	2.82
**0.60**	−20.39	−12.64	−8.96	−5.36	−1.91	**9**	2.45	2.52	2.63	2.77	2.87
**0.80**	−14.53	−8.87	−6.06	−3.28	−0.61	**12**	2.54	2.62	2.70	2.81	2.88
**0.10**	−11.02	−6.63	−4.33	−2.02	0.18	**15**	2.59	2.66	2.74	2.84	2.89
**1.20**	−8.67	−5.11	−3.19	−1.15	0.75	**18**	2.63	2.70	2.77	2.85	2.90
**1.40**	−7.00	−3.95	−2.37	−0.52	1.13	**21**	2.66	2.74	2.80	2.87	2.91
**1.60**	−5.74	−3.08	−1.73	−0.05	1.44	**24**	2.69	2.76	2.82	2.88	2.92
**1.80**	−4.76	−2.36	−1.23	0.33	1.67	**27**	2.72	2.78	2.83	2.88	2.93
**2.00**	−3.98	−1.81	−0.81	0.61	1.86	**30**	2.74	2.80	2.85	2.89	2.94
**[Glycyl Dipeptide] = 0.010 mol∙kg^−1^**											
**0.20**	−52.15	−31.60	−23.37	−18.12	−5.54	**3**	1.55	2.26	2.46	2.51	2.73
**0.40**	−24.55	−14.30	−10.11	−7.58	−1.69	**6**	2.17	2.57	2.59	2.71	2.85
**0.60**	−15.35	−8.52	−5.74	−3.88	−0.75	**9**	2.41	2.67	2.69	2.79	2.88
**0.80**	−10.74	−5.66	−3.55	−2.02	−0.28	**12**	2.52	2.72	2.74	2.82	2.90
**0.10**	−7.99	−3.96	−2.23	−0.92	0.00	**15**	2.61	2.75	2.78	2.84	2.91
**1.20**	−6.17	−2.83	−1.32	−0.17	0.19	**18**	2.66	2.78	2.81	2.86	2.92
**1.40**	−4.87	−1.97	−0.71	0.35	0.40	**21**	2.70	2.80	2.83	2.87	2.92
**1.60**	−3.88	−1.30	−0.23	0.78	0.55	**24**	2.73	2.81	2.84	2.89	2.93
**1.80**	−3.10	−0.78	0.14	1.08	0.67	**27**	2.76	2.83	2.86	2.89	2.93
**2.00**	−2.46	−0.34	0.47	1.32	0.67	**30**	2.77	2.84	2.86	2.90	2.94

Standard uncertainties, u, are u (T) = 0.01 K, u (p) = 10 kPa, u (molarity of glycyl dipeptide) 0.0005 mol·kg^−1^ u (molarity of NaC) = 0.001 mol·kg^−1^, u (molarity of NaDC) = 0.002 mol·kg^−1^ and u (V_φ_) = 0.05 × 10^−6^ m^3^∙mol^−1^.

**Table 4:** Apparent molar isentropic compression, $\kappa_{s,\varphi }$/10^−3^ (m^3^∙mol^−1^∙Pa^−1^) values for CTAB and DTAB in 0.001, 0.005 and 0.010 mol∙kg^−1^ aqueous solution of glycyl dipeptide at different temperatures and pressure *p* = 100 kPa.

CTAB						DTAB					
[CTAB] mmol∙kg^−1^	293.15 K	298.15 K	303.15 K	308.15 K	313.15 K	[DTAB] mmol∙kg^−1^	293.15 K	298.15 K	303.15 K	308.15 K	313.15 K
**[Pure Water]**											
**0.20**	−1.53	−1.36	−1.21	−1.05	−0.68	**3**	−0.09	−0.06	−0.02	−0.01	0.00
**0.40**	−0.90	−0.79	−0.65	−0.56	−0.44	**6**	−0.07	−0.04	−0.01	0.00	0.01
**0.60**	−0.72	−0.66	−0.46	−0.40	−0.32	**9**	−0.06	−0.03	−0.01	0.00	0.02
**0.80**	−0.59	−0.54	−0.38	−0.31	−0.26	**12**	−0.05	−0.02	−0.01	0.00	0.02
**1.00**	−0.55	−0.46	−0.30	−0.25	−0.22	**15**	−0.04	−0.02	0.00	0.01	0.02
**1.20**	−0.47	−0.39	−0.24	−0.20	−0.17	**18**	−0.02	−0.01	0.02	0.02	0.03
**1.40**	−0.42	−0.35	−0.21	−0.17	−0.14	**21**	0.01	0.01	0.03	0.04	0.05
**1.60**	−0.38	−0.31	−0.18	−0.14	−0.12	**24**	0.01	0.02	0.04	0.05	0.06
**1.80**	−0.34	−0.27	−0.15	−0.12	−0.10	**27**	0.02	0.03	0.05	0.06	0.07
**2.00**	−0.32	−0.25	−0.14	−0.11	−0.08	**30**	0.03	0.04	0.06	0.07	0.07
**[Glycyl Dipeptide] = 0.001 mol∙kg^−1^**											
**0.20**	−70.32	−58.42	−50.25	−44.92	−33.01	**3**	−3.53	−2.96	−2.33	−1.95	−1.20
**0.40**	−35.75	−30.56	−26.13	−22.84	−16.79	**6**	−1.57	−1.25	−0.93	−0.47	−0.32
**0.60**	−24.25	−20.94	−17.76	−15.22	−11.37	**9**	−1.08	−0.75	−0.52	−0.21	−0.18
**0.80**	−18.59	−15.87	−13.38	−11.38	−8.56	**12**	−0.88	−0.58	−0.37	−0.13	−0.08
**0.10**	−15.06	−13.10	−10.79	−9.08	−6.84	**15**	−0.71	−0.46	−0.26	−0.03	0.01
**1.20**	−12.71	−11.10	−9.05	−7.48	−5.75	**18**	−0.48	−0.28	−0.10	0.09	0.13
**1.40**	−11.03	−9.49	−7.75	−6.38	−4.94	**21**	−0.35	−0.10	0.04	0.22	0.21
**1.60**	−9.77	−8.39	−6.76	−5.55	−4.28	**24**	−0.17	0.01	0.18	0.33	0.31
**1.80**	−8.76	−7.52	−5.99	−4.90	−3.78	**27**	−0.09	0.15	0.28	0.41	0.40
**2.00**	−8.01	−6.79	−5.36	−4.36	−3.34	**30**	0.03	0.26	0.37	0.49	0.48
**[Glycyl Dipeptide] = 0.005 mol∙kg^−1^**											
**0.20**	−93.33	−69.34	−35.68	−21.53	−17.12	**3**	−3.23	−2.77	−1.97	−1.68	−1.31
**0.40**	−47.59	−35.03	−18.61	−11.22	−9.11	**6**	−1.43	−1.19	−0.71	−0.45	−0.18
**0.60**	−31.96	−23.89	−13.01	−7.80	−6.19	**9**	−0.99	−0.82	−0.52	−0.33	−0.05
**0.80**	−24.44	−18.29	−10.37	−6.04	−4.83	**12**	−0.84	−0.65	−0.34	−0.22	0.06
**0.10**	−20.05	−14.77	−8.40	−5.08	−3.88	**15**	−0.66	−0.48	−0.22	−0.13	0.16
**1.20**	−16.88	−12.42	−7.18	−4.37	−3.32	**18**	−0.45	−0.28	−0.07	0.03	0.25
**1.40**	−14.78	−10.70	−6.32	−3.77	−2.93	**21**	−0.26	−0.09	0.06	0.19	0.36
**1.60**	−13.13	−9.34	−5.68	−3.36	−2.58	**24**	−0.10	0.04	0.18	0.31	0.46
**1.80**	−12.02	−8.31	−5.15	−3.03	−2.33	**27**	0.01	0.17	0.28	0.40	0.54
**2.00**	−10.29	−7.46	−4.65	−2.70	−2.20	**30**	0.11	0.26	0.37	0.47	0.60
**[Glycyl Dipeptide] = 0.010 mol∙kg^−1^**											
**0.20**	−52.58	−40.44	−35.93	−33.22	−16.62	**3**	−2.12	−1.56	−1.30	−1.01	−0.48
**0.40**	−26.34	−20.29	−18.11	−16.83	−9.01	**6**	−1.24	−0.92	−0.74	−0.37	−0.22
**0.60**	−17.59	−13.65	−12.21	−11.21	−6.77	**9**	−0.91	−0.66	−0.50	−0.23	−0.10
**0.80**	−13.13	−10.30	−9.19	−8.32	−5.57	**12**	−0.79	−0.47	−0.38	−0.12	−0.03
**0.10**	−10.48	−8.23	−7.31	−6.54	−4.80	**15**	−0.56	−0.33	−0.24	−0.02	0.10
**1.20**	−8.66	−6.81	−6.04	−5.30	−4.24	**18**	−0.35	−0.15	−0.05	0.14	0.21
**1.40**	−7.37	−5.75	−5.12	−4.42	−3.74	**21**	−0.15	0.01	0.10	0.25	0.33
**1.60**	−6.39	−4.90	−4.37	−3.73	−3.36	**24**	0.00	0.14	0.22	0.34	0.43
**1.80**	−5.61	−4.23	−3.82	−3.22	−3.07	**27**	0.11	0.25	0.32	0.42	0.51
**2.00**	−4.95	−3.68	−3.36	−2.79	−2.92	**30**	0.19	0.33	0.40	0.54	0.58

Standard uncertainties, u, are u (T) = 0.01 K, u (p) = 10 kPa, u (molatity of glycyl dipeptide) 0.0005 mol·kg^−1^ u (molarity of NaC) = 0.001 mol·kg^−1^, u (molarity of NaDC) = 0.002 mol·kg^−1^ and u (κ_s,φ_) = 0.05 × 10^−3^ m^3^∙mol^−1^∙TPa^−1^.

#### Effect of Glycyl Dipeptide

In this section, we report the variation of $\left(V_{\phi }\right)$ and $\left(\kappa_{S,\phi }\right)$ values for CTAB and DTAB in aqueous solutions of glycyl dipeptide for the concentrations 0.001, 0.005 and 0.010 mol kg^−1^ at different temperatures ranging from 293.15 K to 313.15 K at a gap of 5 K. The data has been analyzed to extract information pertaining to the involvement of glycyl dipeptide in upsetting the surfactant–dipeptide interactions in aqueous medium. However, in order to discuss the behavior of apparent molar volume $\left(V_{\phi }\right)$ and apparent molar isentropic compression $\left(\kappa_{S,\phi }\right)$ it is imperative to have some basic knowledge of compressibility of the solution. Although it does not furnish a precise scheme of the interaction pattern pertained in the system, it certainly establishes the proximity of various kinds of interactions existing in the system. The compressibility $\left(\kappa_{S,\phi }\right)$ of micellar solutions has been proposed to mainly depend on:

(i) ease with which a non-polar micellar core can be compressed, and

(ii) nature of interactions among polar head groups of the surfactants and solute.

Moreover, isentropic compressibility is reliant on the alteration of the counter ion bound to the polar head of the surfactants and the hydrophilicity of the surfactant head group, i.e., polar head groups, present on the surfactant. The compressibility data (Table 4), illustrate that the isentropic compressibility $\left(\kappa_{S}\right)$ decreases with [CTAB/DTAB] as well as with temperature [37], which signifies that the solution becomes more difficult to compress. This decrease in compressibility and, consequently, increase in speed in sound has been accredited to the fact that solute and solvent interacts strongly as concentration and temperature increases, which may be due to formation of hydrogen bonds. Moreover, the value of $\left(\kappa_{S,\phi }\right)$ also decreased with increase in the content of glycyl dipeptide. As we know, the glycyl dipeptide molecules in the neutral solution exist in zwitterionic form and thus strongly interact with the surrounding water molecules (polar). This increases theelectrostrictive compression by the aqueous environment in proximity the solute molecules, resulting in less compressibility of the solution [38]. Some specific interactions including hydrophobic and electrostatic interactions have been anticipated to exist in the CTAB/DTAB + glycyl dipeptide system at low CTAB/DTAB concentration, which shifts to some non-specific or cooperative interaction types at higher concentrations [39]. Taking thisevidence into consideration, both hydrophilic and hydrophobic interactions among surfactants and glycyl dipeptide are likely to contribute in complex formation.

**Figure 3:**
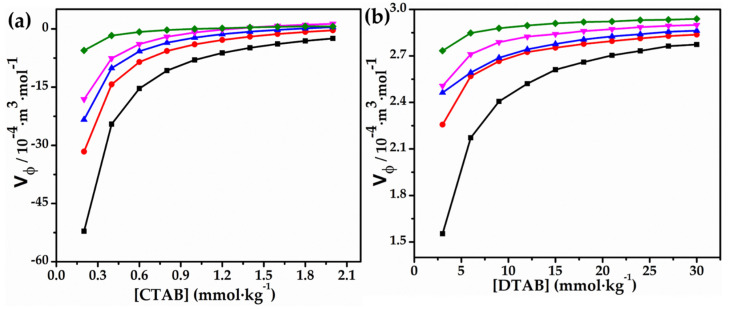
Plots of $V_{\varphi }$ versus (**a**) [CTAB] and (**b**) [DTAB] in 0.010 mol∙kg^−1^ aqueous solution of glycyl dipetide293.15 K (■), 298.15 K (●), 303.15 K (▲), 308.15 K (▼) and 313.15 K (◆).

**Figure 4:**
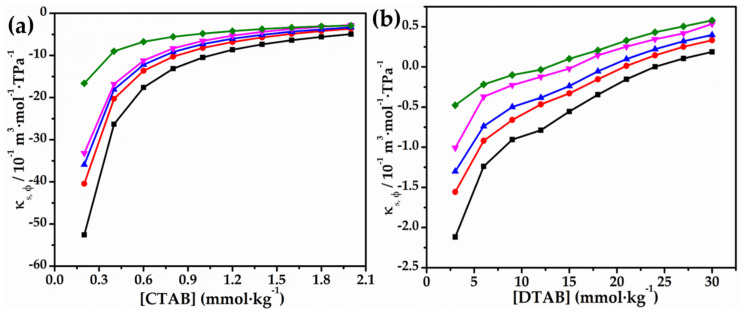
Plots of $\kappa_{s,\phi }$ versus (**a**) [CTAB] and (**b**) [DTAB] in 0.010 mol∙kg^−1^ aqueous solution of glycyl dipetide293.15 K (■), 298.15 K (●), 303.15 K (▲), 308.15 K (▼) and 313.15 K (◆).

On the other hand, apparent molar volume $\left(V_{\phi }\right)$ values of CTAB and DTAB tend to increase upon the addition of glycyl dipeptide, as can be observed from Table 3. This observation supports the fact that the intermolecular interactions of the considered surfactants (CTAB and DTAB) with dipeptide give rise to a complex formation which has greater hydrophobic character. The values of $\left(V_{\phi }\right)$ and $\left(\kappa_{S,\phi }\right)$ as a function of surfactant content havebeen graphically presented in Figure 3 and Figure 4, respectively. Numerous reports on solution behavior of non-polar solutes direct towards the effect of temperature on the volumetric characteristics of such molecules, which are conferred as hydrational modifications. In solutions containing electrolytes (hydrophilic), information regarding solute–solute interactions may be grasped from positive slopes of $\left(V_{\phi }\right)$ versus concentration plots. The positive slopes of such graphs signify destructive overlap of co-sphere which results in a net diminishment of solvation. Conversely, in solution containing non-electrolyte (hydrophobic) solutes, the slopes of these plots turn out to be more negative, and become more negative (decrease) with the hydrophobicity of the solute molecule. The negative slopes of $\left(V_{\phi }\right)$ versus concentration plots inform us about the solute–solute interactions, and are recommended to be the consequence of constructive overlap of the co-spheres which lead to a net increase in salvation. Consequently, the total apparent molar volume of solute decreases. In these situations, it is crucial to point out that $V_{\phi }$ CTAB values exhibit little to no concentration dependence, suggesting a balance between their hydrophilic and hydrophobic contributions. Furthermore, it has been shown that the systems under consideration exhibit strong solute–solvent interactions, which become stronger as temperature increases. A pattern shown in Figure 3 for $\left(V_{\phi }\right)$ has a curved shape at low surfactant concentrations and becomes linear or slightly curved at higher surfactant concentrations [40]. Additionally, in the presence of glycyl dipeptide, apparent molar volume $\left(V_{\phi }\right)$ exhibits a strong concentration-dependent relationship with CTAB and DTAB, which amplifies the hydrophobic hydration of the surfactant molecules and changes the solvent–solvent interactions. The formation of complex aggregates of CTAB/DTAB and glycyl dipeptide may be interpreted as the result of interactions between the cationic surfactant and glycyl dipeptide, as suggested by the change in the critical micelle concentration (CMC), or the minimum concentration of surfactant at which micelles are formed, region of these surfactants.

The apparent molar isentropic compression $\left(\kappa_{S,\phi }\right)$ of CTAB and DTAB in aqueous solutions of glycyl dipeptide also appears to be of great importance with respect to the interactions between surfactant and dipeptide. From Figure 4, it can be inferred that the $\left(\kappa_{S,\phi }\right)$ value with [CTAB/DTAB] seem to be almost similar at all temperatures and concentrations of glycyl dipeptide. Therefore, it advocates that the modifications that occur at a low concentration of surfactants are consistent with the observation that characterizes the surfactant–glycyl dipeptide interactions resulting into the formation of “clathrate-like” structures of surfactant/glycyl dipeptide. Since $\kappa_{S,\phi }$ values are negative for both CTAB and DTAB, it recommends that there is the existence of breakdown in solvent (water in present case) structure as well. Nevertheless, at higher CTAB and DTAB content, as $\kappa_{S,\phi }$ values are less negative, it seems that the surfactant affects the glycyl dipeptide molecules and encourages them to occupy the micellar interior and get solubilized in the micellar region, accompanying the increase in free space. Additionally, from data reported in Table 4, for $\kappa_{S,\phi }$, it can be observed that the $\left(\kappa_{S,\phi }\right)$ value of DTAB changes sign (negative to positive) with an increase in surfactant concentration, which is important in understanding the difference in the solution behavior of surfactants in aqueous solutions of glycyl dipeptide. Therefore, it can be suggested that hydrophilic hydration of CTAB-glycyl dipeptide complex occurs at a low surfactant concentration region, rendering $\left(\kappa_{S,\phi }\right)$ < 0. The negative $\left(\kappa_{S,\phi }\right)$ values observed to increase, i.e., become less negative (even positive for DTAB), with CTAB/DTAB concentration is consequently because of loss of hydrophobic hydration of surfactant–glycyl dipeptide complex. The alteration in the behavior of surfactants towards apparent molar isentropic compression may be due to the cooperative self-aggregation of surfactants at certain concentrations, i.e., micelle formation. As a result, we can infer that the rise in the surfactant $\left(\kappa_{S,\phi }\right)$ value with temperature is likewise caused by the dehydration of hydrophobic molecules. These findings are discovered to be quite similar to other studies that have been published in the literature [41,42].

### 3.2. Viscometric Studies

This section explicates the information grasped from viscosity measurements of CTAB and DTAB with glycyl dipeptide and an effort has been made to investigate the interactions between these surface active agents with different additives in aqueous medium under varying experimental conditions. Monitoring all processes that result in intermolecular interactions and arrangement of the molecules in the system is crucial [22,43,44,45] because solution parameters like viscosity are sensitive to molecular structure [43,44,45]. In Table S3 of the Supplementary Materials, the viscosity data of the cationic surfactants CTAB and DTAB in aqueous solutions of glycyl dipeptide are presented. Viscosity values have been calculated by using the given equation:

(3) $$\eta =\eta_{o}\frac{\rho \times t}{\rho_{o}\times t_{o}}$$

where $\eta_{o}$, $t_{o}$ and $\rho_{o}$ are the viscosity, time of flow and density of the solvent system, respectively, and $\eta_{}$, t and ρ are the viscosity, time of flow and density of the solution, respectively. On an inspection of viscosity data, it has been found that the η values increase with rise in concentration of both the cationic surfactant as well as with the concentrations of glycyl dipeptide, which may be ascribed to intermolecular interactions existing in the solution that are electrostatic as well as hydrophobic in nature. Captivatingly, viscosity values show noteworthy variation within the concentration range near to CMC for both the cationic surfactants. This type of behavior gives confirmation of structural switches due to the micellization process of these cationic surfactants at the CMC range in the aqueous glycyl dipeptide solutions. However, when the temperature rises, the η values show a decline. This is attributed to fact that the intermolecular interactions decrease because of an increase in kinetic energy of the species present, thereby decreasing the viscous force [46,47,48]. For the cationic surfactants used in the present study the viscosity values vary in the order: CTAB > DTAB, which is as expected by the greater hydrophobic character of CTAB and thereby facilitates micellization/aggregation to greater extent [25].

Furthermore, viscosity data have also been subjected to calculate relative viscosity, $\eta_{r}$ by using following the equation [49].

(4) $$\eta_{r}=\frac{\eta }{\eta_{0}}$$

**Figure 5:**
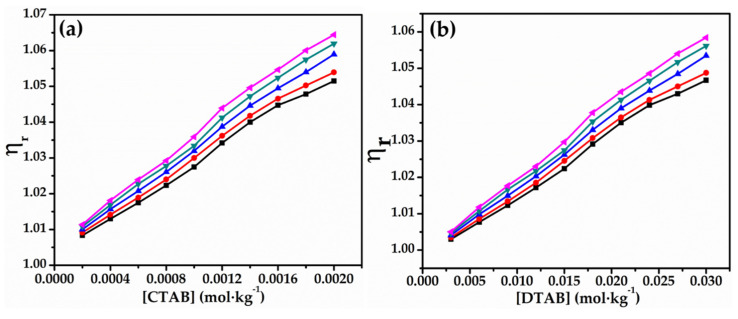
Plots of $\eta_{r}$ versus (**a**) [CTAB] and (**b**) in 0.010 mol∙kg^−1^ aqueous solutions of glycyl dipeptide at 293.15 K (■), 298.15 K (●), 303.15 K (▲), 308.15 K (▼) and 313.15 K (◆).

The values of relative viscosity have been placed in Table 5, and the plots of relative viscosity versus concentration of both the surfactants in 0.010 mol∙kg^−1^ of glycyl dipeptide in aqueous solution are shown in Figure 5. It has been noted from the plots that relative viscosity shows a steady increase at lower concentrations of both the cationic surfactants (<0.8 mmol∙kg^−1^ for CTAB and <13 mmol∙kg^−1^ for DTAB), but escalate abruptly at higher concentrations [50,51]. Interestingly, this observation of results on relative viscosity has been consistent with the above discussed variations in viscosity measurements as well.

The viscosity data, in combination with density and speed of sound values, have also been used to estimate viscous relaxation time (τ), which play an important role to provide qualitative data regarding the nature and strength of intermolecular interactions persisting in the liquid mixtures [52,53]. Viscous relaxation time (τ) for these cationic surfactants in aqueous solutions of glycyl dipeptide at different temperatures have been indexed in Table 6 and estimated by using the equation given below [54]:

$$\tau =\frac{4}{3}\frac{\eta }{u^{2}\rho }$$

where η, ρ and u are the viscosity, density and speed of sound of the solution.

The viscous relaxation time, which is dependent on temperature and impurities and is used to gather details about numerous intermolecular interactions in the system, is the amount of time it takes for the excitation energy to manifest as translational energy [55]. Because of this, it is also supported by the fact that its temperature sensitivity andstructural relaxation processes are caused by the instantaneous conversion of excitation energy to translational energy. It has been recorded that τ values with concentration of surfactant vary in a similar fashion to relative viscosity $\eta_{r}$ of the studied system. From the data on viscous relaxation time, it has been clearly seen that τ values rise gradually on increasing the concentrations of both the surfactants used, as well as with concentration of glycyl dipeptide, and fall off with temperature. This occurs mostly as a result of structural relaxation processes that take place during the rearrangement of the molecules in the system under study. Moreover, the τ values for cationic surfactants have been found to be of higher magnitude for CTAB (within experimental error) respectively, in aqueous solutions of glycyl dipeptide. These results strengthen the results obtained from earlier studies [56] on micellization and clearly reflect the structural relationship, i.e., the more hydrophobic nature of surfactants.

## 4. Conclusions

In light of the various parameters evaluated and examined thoroughly in this paper, the following conclusions have been drawn:

(i) The interactions between CTAB/DTAB and glycyl dipeptide have been found to be concentration dependent. The nature of interactions show remarkable changes in the micellar region of these surfactants, i.e., shifts from the hydrophilic to hydrophobic kind, and leads to the formation of complex aggregates of CTAB/DTAB and glycyl dipeptide.
(ii) A substantial decrease has also been observed in the apparent molar volume and compressibility of the monomer with respect to its value in water. Both apparent molar volume and isentropic compressibility have been found to follow the same trend at higher surfactant concentrations for the surfactants, i.e., CTAB and DTAB.
(iii) τ values and relative viscosity $\eta_{r}$ values also show concentration and temperature dependence; however, the results are more pronounced with CTAB than that of DTAB in aqueous solutions of glycyl dipeptide.

## Figures and Tables

**Table 1:** Specification of chemicals used.

Chemical Name	Source	Mol.Wt./kg∙mol^−1^	Purification Method	Mass Fraction Purity ^a^
Glycylglycine	Spectrochem Pvt. Ltd. (New Delhi, India)	0.132	None	0.98
Cetyltrimethylammonium Bromide	Himedia Pvt. Ltd. (Mumbai, India)	0.364	Recrystallization	0.98
Dodecyltrimethylammonium Bromide	Himedia Pvt. Ltd. (Mumbai, India)	0.308	Recrystallization	0.98

^a^ Declared by the supplier.

**Table 2:** Isentropic compressibility, $\kappa_{s}$ (TPa^−1^) values for CTAB and DTAB in pure water and in 0.001, 0.005 and 0.010 mol∙kg^−1^ aqueous solution of glycyldipeptide at different temperatures and pressure *p* = 100 kPa.

CTAB						DTAB					
[CTAB] mmol∙kg^−1^	293.15 K	298.15 K	303.15 K	308.15 K	313.15 K	[DTAB] mmol∙kg^−1^	293.15 K	298.15 K	303.15 K	308.15 K	313.15 K
**[Pure Water]**											
**0.2**	455.79	447.77	440.89	435.50	431.13	**3**	455.27	447.34	440.64	435.17	430.87
**0.4**	455.71	447.70	440.84	435.46	431.07	**6**	454.72	446.91	440.25	434.79	430.55
**0.6**	455.61	447.59	440.80	435.41	431.03	**9**	454.23	446.48	439.87	434.44	430.25
**0.8**	455.54	447.52	440.74	435.37	430.98	**12**	453.76	446.07	439.45	434.10	429.94
**1.0**	455.42	447.46	440.72	435.34	430.94	**15**	453.33	445.65	439.20	433.78	429.65
**1.2**	455.37	447.42	440.69	435.32	430.92	**18**	453.30	445.43	439.10	433.71	429.57
**1.4**	455.32	447.38	440.67	435.30	430.90	**21**	453.28	445.38	439.08	433.71	429.58
**1.6**	455.27	447.34	440.64	435.28	430.88	**24**	453.05	445.33	439.06	433.70	429.59
**1.8**	455.23	447.32	440.62	435.25	430.86	**27**	452.94	445.27	439.04	433.70	429.60
**2.0**	455.17	447.27	440.59	435.23	430.84	**30**	452.87	445.23	439.02	433.72	429.62
**[Glycyl Dipeptide] = 0.001 mol∙kg^−1^**											
**0.20**	447.20	440.57	435.09	431.55	427.61	**3**	454.32	446.41	439.90	434.46	430.31
**0.40**	447.15	440.50	435.03	431.51	427.57	**6**	454.02	446.14	439.62	434.34	430.08
**0.60**	447.10	440.44	434.98	431.49	427.54	**9**	453.58	445.76	439.29	434.06	429.73
**0.80**	447.05	440.40	434.96	431.47	427.51	**12**	453.07	445.34	438.95	433.72	429.43
**0.10**	447.00	440.33	434.92	431.45	427.49	**15**	452.68	444.96	438.62	433.46	429.16
**1.20**	446.96	440.28	434.89	431.43	427.47	**18**	452.48	444.76	438.44	433.28	428.99
**1.40**	446.92	440.26	434.87	431.41	427.45	**21**	452.28	444.67	438.33	433.18	428.83
**1.60**	446.87	440.22	434.85	431.39	427.43	**24**	452.19	444.54	438.28	433.13	428.74
**1.80**	446.83	440.18	434.82	431.37	427.41	**27**	452.02	444.51	438.24	433.08	428.70
**2.00**	446.79	440.15	434.80	431.36	427.40	**30**	451.93	444.48	438.19	433.04	428.67
**[Glycyl Dipeptide] = 0.005 mol∙kg^−1^**											
**0.20**	446.65	440.08	434.30	430.37	427.04	**3**	454.14	446.27	439.83	434.32	430.08
**0.40**	446.59	440.05	434.25	430.33	427.00	**6**	453.84	445.98	439.59	434.16	429.75
**0.60**	446.55	440.00	434.19	430.28	426.96	**9**	453.39	445.55	439.17	433.77	429.43
**0.80**	446.48	439.95	434.12	430.24	426.92	**12**	452.89	445.13	438.85	433.42	429.17
**0.10**	446.41	439.91	434.09	430.19	426.89	**15**	452.54	444.81	438.55	433.12	428.97
**1.20**	446.36	439.88	434.04	430.15	426.85	**18**	452.34	444.64	438.36	432.98	428.79
**1.40**	446.29	439.84	434.00	430.12	426.81	**21**	452.22	444.56	438.22	432.94	428.72
**1.60**	446.23	439.82	433.95	430.08	426.78	**24**	452.12	444.47	438.15	432.89	428.68
**1.80**	446.14	439.79	433.91	430.04	426.74	**27**	451.99	444.41	438.10	432.85	428.64
**2.00**	446.22	439.77	433.88	430.02	426.69	**30**	451.90	444.35	438.04	432.81	428.61
**[Glycyl Dipeptide] = 0.010 mol∙kg^−1^**											
**0.20**	445.41	439.02	433.83	429.00	425.81	**3**	453.93	446.27	439.80	434.25	430.04
**0.40**	445.38	438.99	433.80	428.96	425.76	**6**	453.69	445.80	439.39	433.90	429.67
**0.60**	445.35	438.95	433.77	428.94	425.70	**9**	453.23	445.38	439.01	433.53	429.34
**0.80**	445.33	438.92	433.74	428.91	425.66	**12**	452.71	445.03	438.62	433.21	429.01
**0.10**	445.30	438.90	433.72	428.89	425.61	**15**	452.42	444.72	438.34	432.95	428.82
**1.20**	445.28	438.88	433.70	428.88	425.57	**18**	452.24	444.55	438.21	432.86	428.66
**1.40**	445.27	438.86	433.68	428.87	425.55	**21**	452.14	444.46	438.13	432.75	428.60
**1.60**	445.25	438.86	433.67	428.86	425.52	**24**	452.07	444.39	438.07	432.66	428.57
**1.80**	445.23	438.85	433.65	428.85	425.49	**27**	451.94	444.33	438.01	432.58	428.52
**2.00**	445.22	438.84	433.64	428.84	425.45	**30**	451.83	444.26	437.96	432.67	428.51

Standard uncertainties, u, are u (T) = 0.01 K, u (p) = 10 kPa, u (molarity of glycyl dipeptide) 0.0005 mol·kg^−1^ u (molarity of NaC) = 0.001 mol·kg^−1^, u (molarity of NaDC) = 0.002 mol·kg^−1^ and u (κ_s_) = 0.21 TPa^−1^.

**Table 5:** Relative viscosity, $\eta_{r}$ values for CTAB and DTAB in pure water and 0.001, 0.005 and 0.010 mol∙kg^−1^ aqueous solution of glycyl dipeptide at different temperatures and pressure *p* = 100 kPa.

CTAB						DTAB					
[CTAB] mmol∙kg^−1^	293.15 K	298.15 K	303.15 K	308.15 K	313.15 K	[DTAB] mmol∙kg^−1^	293.15 K	298.15 K	303.15 K	308.15 K	313.15 K
**[Pure water]**											
**0.2**	1.0042	1.0056	1.0066	1.0085	1.0096	**3**	1.002	1.004	1.005	1.006	1.007
**0.4**	1.0087	1.0103	1.0121	1.0147	1.0165	**6**	1.004	1.007	1.008	1.010	1.012
**0.6**	1.0136	1.0154	1.0171	1.0206	1.0226	**9**	1.006	1.010	1.012	1.014	1.016
**0.8**	1.0181	1.0201	1.0223	1.0260	1.0284	**12**	1.008	1.012	1.014	1.017	1.019
**1.0**	1.0228	1.0250	1.0274	1.0316	1.0342	**15**	1.010	1.014	1.016	1.020	1.022
**1.2**	1.0275	1.0299	1.0322	1.0369	1.0394	**18**	1.012	1.017	1.019	1.023	1.025
**1.4**	1.0320	1.0347	1.0371	1.0420	1.0446	**21**	1.015	1.020	1.022	1.026	1.028
**1.6**	1.0361	1.0390	1.0418	1.0468	1.0499	**24**	1.019	1.024	1.026	1.030	1.033
**1.8**	1.0409	1.0440	1.0463	1.0520	1.0549	**27**	1.021	1.026	1.028	1.032	1.035
**2.0**	1.0456	1.0489	1.0517	1.0564	1.0600	**30**	1.025	1.030	1.032	1.037	1.040
**[Glycyl Dipeptide] = 0.001 mol∙kg^−1^**											
**0.20**	1.014	1.014	1.006	1.010	1.012	**3**	0.864	0.867	0.882	0.896	0.858
**0.40**	1.028	1.026	1.020	1.026	1.027	**6**	0.869	0.873	0.887	0.899	0.861
**0.60**	1.039	1.037	1.038	1.042	1.045	**9**	0.875	0.898	0.891	0.905	0.865
**0.80**	1.050	1.053	1.053	1.056	1.061	**12**	0.881	0.884	0.896	0.910	0.868
**0.10**	1.064	1.069	1.068	1.075	1.080	**15**	0.886	0.890	0.899	0.914	0.874
**1.20**	1.075	1.077	1.086	1.090	1.098	**18**	0.890	0.896	0.902	0.919	0.878
**1.40**	1.084	1.088	1.103	1.105	1.114	**21**	0.897	0.902	0.908	0.920	0.883
**1.60**	1.093	1.101	1.119	1.121	1.128	**24**	0.901	0.908	0.910	0.924	0.884
**1.80**	1.101	1.117	1.133	1.138	1.143	**27**	0.907	0.912	0.914	0.925	0.889
**2.00**	1.113	1.134	1.148	1.158	1.164	**30**	0.911	0.918	0.918	0.928	0.891
**[Glycyl Dipeptide] = 0.005 mol∙kg^−1^**											
**0.20**	1.014	1.017	1.013	1.007	1.013	**3**	0.865	0.868	0.877	0.881	0.858
**0.40**	1.023	1.030	1.028	1.022	1.030	**6**	0.872	0.874	0.882	0.885	0.864
**0.60**	1.032	1.041	1.042	1.038	1.051	**9**	0.875	0.880	0.886	0.891	0.869
**0.80**	1.041	1.055	1.057	1.050	1.070	**12**	0.879	0.886	0.890	0.894	0.872
**0.10**	1.052	1.066	1.071	1.068	1.085	**15**	0.883	0.892	0.894	0.899	0.877
**1.20**	1.063	1.077	1.084	1.082	1.099	**18**	0.888	0.897	0.897	0.900	0.879
**1.40**	1.074	1.088	1.100	1.101	1.118	**21**	0.893	0.902	0.903	0.903	0.882
**1.60**	1.082	1.102	1.113	1.114	1.132	**24**	0.899	0.906	0.907	0.905	0.887
**1.80**	1.096	1.116	1.128	1.131	1.148	**27**	0.905	0.913	0.911	0.908	0.891
**2.00**	1.108	1.130	1.153	1.144	1.165	**30**	0.908	0.918	0.913	0.910	0.894
**[Glycyl Dipeptide] = 0.010 mol∙kg^−1^**											
**0.20**	1.011	1.010	1.013	1.013	1.007	**3**	0.868	0.879	0.889	0.865	0.853
**0.40**	1.021	1.023	1.034	1.025	1.021	**6**	0.879	0.887	0.895	0.872	0.860
**0.60**	1.035	1.032	1.050	1.041	1.035	**9**	0.888	0.893	0.901	0.877	0.865
**0.80**	1.050	1.046	1.067	1.053	1.052	**12**	0.895	0.899	0.906	0.883	0.870
**0.10**	1.058	1.059	1.091	1.069	1.066	**15**	0.901	0.903	0.912	0.887	0.875
**1.20**	1.069	1.072	1.109	1.084	1.073	**18**	0.910	0.909	0.917	0.894	0.880
**1.40**	1.081	1.086	1.124	1.096	1.086	**21**	0.913	0.915	0.922	0.897	0.884
**1.60**	1.093	1.099	1.141	1.109	1.100	**24**	0.916	0.918	0.927	0.902	0.889
**1.80**	1.104	1.110	1.159	1.121	1.114	**27**	0.919	0.921	0.933	0.907	0.892
**2.00**	1.114	1.119	1.175	1.133	1.128	**30**	0.924	0.924	0.938	0.909	0.898

Standard uncertainties, u, are u (T) = 0.01 K, u (p) = 10 kPa, u (molarity of glycyl dipeptide) 0.0005 mol·kg^−1^ u (molarity of NaC) = 0.001 mol·kg^−1^, u (molarity of NaDC) = 0.002 mol·kg^−1^ and u (η_r_) = 0.020 mPa∙s^−1^.

**Table 6:** Viscous relaxation time, τ (ps) values for CTAB and DTAB in pure water and 0.001, 0.005 and 0.010 mol∙kg^−1^ aqueous solution of glycyl dipeptide at different temperaturesand pressure *p* = 100 kPa.

CTAB						DTAB					
[CTAB] mmol∙kg^−1^	293.15 K	298.15 K	303.15 K	308.15 K	313.15 K	[DTAB] mmol∙kg^−1^	293.15 K	298.15 K	303.15 K	308.15 K	313.15 K
**[Water]**											
**0.2**	0.6110	0.5350	0.4720	0.4210	0.3550	**3**	0.609	0.533	0.471	0.420	0.354
**0.4**	0.6140	0.5370	0.4740	0.4240	0.3580	**6**	0.610	0.534	0.472	0.421	0.356
**0.6**	0.6170	0.5400	0.4770	0.4260	0.3600	**9**	0.610	0.535	0.473	0.422	0.357
**0.8**	0.6200	0.5420	0.4790	0.4290	0.3620	**12**	0.611	0.536	0.474	0.423	0.358
**1.0**	0.6220	0.5440	0.4810	0.4310	0.3640	**15**	0.612	0.537	0.475	0.424	0.359
**1.2**	0.6250	0.5470	0.4840	0.4330	0.3660	**18**	0.613	0.538	0.476	0.425	0.360
**1.4**	0.6280	0.5490	0.4860	0.4350	0.3680	**21**	0.614	0.539	0.477	0.427	0.361
**1.6**	0.6300	0.5520	0.4880	0.4370	0.3690	**24**	0.617	0.541	0.479	0.429	0.362
**1.8**	0.6330	0.5540	0.4900	0.4390	0.3710	**27**	0.618	0.542	0.480	0.430	0.363
**2.0**	0.6360	0.5570	0.4930	0.4410	0.3730	**30**	0.620	0.544	0.482	0.431	0.365
**[Glycyl Dipeptide] = 0.001 mol∙kg^−1^**											
**0.20**	0.850	0.747	0.656	0.619	0.526	**3**	0.715	0.649	0.623	0.605	0.555
**0.40**	0.861	0.755	0.664	0.629	0.534	**6**	0.719	0.653	0.626	0.606	0.557
**0.60**	0.870	0.763	0.676	0.639	0.543	**9**	0.724	0.671	0.628	0.610	0.558
**0.80**	0.880	0.775	0.686	0.647	0.551	**12**	0.728	0.660	0.632	0.613	0.560
**0.10**	0.891	0.786	0.696	0.659	0.562	**15**	0.731	0.663	0.633	0.615	0.563
**1.20**	0.900	0.792	0.707	0.668	0.571	**18**	0.734	0.668	0.635	0.618	0.566
**1.40**	0.908	0.800	0.718	0.677	0.579	**21**	0.740	0.672	0.639	0.619	0.569
**1.60**	0.915	0.810	0.729	0.687	0.586	**24**	0.743	0.676	0.641	0.621	0.570
**1.80**	0.922	0.822	0.738	0.697	0.594	**27**	0.747	0.679	0.643	0.622	0.572
**2.00**	0.932	0.834	0.747	0.709	0.605	**30**	0.750	0.684	0.646	0.624	0.574
**[Glycyl Dipeptide] = 0.005 mol∙kg^−1^**											
**0.20**	0.869	0.760	0.671	0.634	0.540	**3**	0.733	0.665	0.642	0.619	0.567
**0.40**	0.876	0.770	0.681	0.644	0.549	**6**	0.738	0.669	0.646	0.622	0.570
**0.60**	0.884	0.778	0.690	0.653	0.560	**9**	0.740	0.673	0.648	0.625	0.573
**0.80**	0.891	0.788	0.700	0.661	0.570	**12**	0.742	0.677	0.650	0.627	0.575
**0.10**	0.901	0.796	0.709	0.672	0.578	**15**	0.745	0.681	0.653	0.630	0.578
**1.20**	0.910	0.805	0.717	0.681	0.585	**18**	0.749	0.684	0.655	0.630	0.579
**1.40**	0.920	0.813	0.728	0.693	0.596	**21**	0.753	0.688	0.659	0.633	0.581
**1.60**	0.926	0.823	0.736	0.701	0.603	**24**	0.759	0.691	0.662	0.634	0.584
**1.80**	0.938	0.833	0.746	0.712	0.611	**27**	0.763	0.696	0.665	0.636	0.587
**2.00**	0.948	0.844	0.763	0.720	0.620	**30**	0.765	0.700	0.666	0.637	0.588
**[Glycyl Dipeptide] = 0.010 mol∙kg^−1^**											
**0.20**	0.875	0.771	0.684	0.647	0.556	**3**	0.751	0.693	0.673	0.625	0.576
**0.40**	0.884	0.781	0.698	0.655	0.563	**6**	0.760	0.699	0.677	0.630	0.580
**0.60**	0.895	0.788	0.709	0.665	0.571	**9**	0.767	0.703	0.681	0.633	0.583
**0.80**	0.908	0.798	0.721	0.673	0.580	**12**	0.772	0.707	0.685	0.637	0.586
**0.10**	0.916	0.808	0.736	0.682	0.588	**15**	0.777	0.709	0.688	0.640	0.589
**1.20**	0.925	0.818	0.748	0.692	0.591	**18**	0.785	0.714	0.692	0.644	0.593
**1.40**	0.935	0.828	0.758	0.700	0.599	**21**	0.787	0.719	0.696	0.646	0.595
**1.60**	0.946	0.839	0.770	0.708	0.606	**24**	0.789	0.721	0.700	0.650	0.598
**1.80**	0.956	0.847	0.782	0.716	0.614	**27**	0.792	0.723	0.704	0.653	0.600
**2.00**	0.964	0.854	0.793	0.724	0.622	**30**	0.796	0.726	0.708	0.655	0.604

Standard uncertainties, u, are u (T) = 0.01 K, u (p) = 10 kPa, u (molarity of glycyl dipeptide) 0.0005 mol·kg^−1^ u (molarity of NaC) = 0.001 mol·kg^−1^, u (molarity of NaDC) = 0.002 mol·kg^−1^ and u (τ) = 0.01 × 10^−3^ ps.

## Data Availability

The data presented in this study are available on request from the corresponding author.

## Supplementary Materials

The following supporting information can be downloaded at: https://www.mdpi.com/article/10.3390/molecules27248767/s1, Table S1: Density, (kg∙m^−3^) values for CTAB and DTAB in pure water and 0.001, 0.005 and 0.010 mol∙kg^−1^ aqueous solution of glycyl dipeptide at different temperatures.; Table S2: Speed of sound, (m∙s^−1^) values for CTAB and DTAB in pure water and 0.001, 0.005 and 0.010 mol∙kg^−1^ aqueous solution of glycyl dipeptide at different temperatures.; Table S3: Viscosity, (mPa∙s) values for CTAB and DTAB in pure water and 0.001, 0.005 and 0.010 mol∙kg^−1^ aqueous solution of glycyl dipeptide at different temperatures.
