# Supplementary material for: Study on Volumetric, Compressibility and Viscometric Behavior of Cationic Surfactants (CTAB and DTAB) in Aqueous Glycyl Dipeptide: A Thermo-Acoustic Approach
Source: Molecules. 2022 Dec 10;27(24):8767. doi: 10.3390/molecules27248767 (PMC9782549; doi:10.3390/molecules27248767)
Supplement: Supplementary file 1 [file molecules-27-08767-s001.zip › molecules-2056244-supplementary.pdf]

Supplementary Material

Study on Volumetric, Compressibility and Viscometric Behaviour of Cationic Surfactants (CTAB and DTAB) in Aqueous Glycyl dipeptide: Thermo-acoustic Approach

Santosh Kumari [1], Suvarcha Chauhan [1,*], Kuldeep Singh [2], Ahmad Umar [3,4,*,†], Hassan Fouad [5] and M. S. Akhtar [6,7]

[1] Department of Chemistry, Himachal Pradesh University, Summer Hill, Shimla 171005, India
[2] Department of Chemistry, MCM DAV College, Kangra176001, India
[3] Department of Chemistry, College of Science and Arts, and Promising Centre for Sensors and Electronic Devices (PCSED), Najran University, Najran 11001, Saudi Arabia
[4] Department of Materials Science and Engineering, The Ohio State University, Columbus, 43210 OH, USA
[5] Applied Medical Science Department, Community College, King Saud University, P.O. Box 10219, Riyadh 11433, Saudi Arabia
[6] School of Semiconductor and Chemical Engineering, Jeonbuk National University, Jeonju 54896, Republic of Korea
[7] Graduate School of Integrated Energy-AI, Jeonbuk National University, Jeonju 54896, Republic of Korea
* Correspondence: scschauhan19@gmail.com (S.C.); ahmadumar786@gmail.com (A.U.)
† Adjunct Professor at the Department of Materials Science and Engineering, The Ohio State University, Columbus, 43210 OH, USA.

Table S1: Density, ρ (kg·m^{-3}) values for CTAB and DTAB in pure water and 0.001, 0.005 and 0.010 mol·kg^{-1}aqueous solution of glycyl dipeptide at different temperatures.

CTAB						DTAB					
**[CTAB] mmol·kg^{-1}**	**293.15 K**	**298.15 K**	**303.15 K**	**308.15 K**	**313.15 K**	**[DTAB] mmol·kg^{-1}**	**293.15 K**	**298.15 K**	**303.15 K**	**308.15 K**	**313.15 K**
**[Pure Water]**											
**0.2**	998.39	997.23	995.83	994.21	992.37	**3**	998.31	997.20	995.71	994.26	992.29
**0.4**	998.39	997.23	995.83	994.22	992.40	**6**	998.37	997.26	995.76	994.31	992.33
**0.6**	998.40	997.24	995.84	994.23	992.40	**9**	998.43	997.32	995.82	994.36	992.38
**0.8**	998.41	997.25	995.85	994.23	992.41	**12**	998.49	997.37	995.87	994.41	992.42
**1.0**	998.41	997.25	995.85	994.23	992.41	**15**	998.54	997.43	995.92	994.46	992.47
**1.2**	998.41	997.25	995.85	994.23	992.42	**18**	998.60	997.49	995.97	994.51	992.51
**1.4**	998.41	997.25	995.86	994.24	992.42	**21**	998.65	997.53	996.02	994.55	992.55
**1.6**	998.42	997.26	995.86	994.24	992.42	**24**	998.70	997.58	996.07	994.59	992.59
**1.8**	998.42	997.26	995.86	994.24	992.43	**27**	998.75	997.63	996.11	994.63	992.63
**2.0**	998.43	997.26	995.87	994.25	992.43	**30**	998.81	997.67	996.16	994.67	992.67
**[Glycyl Dipeptide] = 0.001 mol·kg^{-1}**											
**0.2**	997.03	996.10	994.63	992.30	990.81	**3**	998.87	997.78	996.32	994.56	992.80
**0.4**	997.11	996.12	994.65	992.32	990.82	**6**	998.88	997.79	996.30	994.53	992.82
**0.6**	997.24	996.13	994.67	992.34	990.84	**9**	998.88	997.69	996.25	994.57	992.85
**0.8**	997.36	996.15	994.69	992.36	990.86	**12**	998.89	997.72	996.36	994.64	992.90
**1.0**	997.45	996.16	994.71	992.37	990.88	**15**	998.96	997.76	996.41	994.71	992.95
**1.2**	997.53	996.18	994.72	992.38	990.91	**18**	999.00	997.81	996.46	994.75	993.00
**1.4**	997.58	996.19	994.74	992.40	990.94	**21**	999.16	997.88	996.51	994.75	993.03
**1.6**	997.62	996.20	994.75	992.42	990.95	**24**	999.20	998.00	996.56	994.82	993.07
**1.8**	997.69	996.21	994.76	992.43	990.97	**27**	999.35	998.00	996.60	994.87	993.10

**2.0**	997.70	996.22	994.78	992.44	990.98	**30**	999.39	998.03	996.65	994.89	993.15
**[Glycyl Dipeptide] = 0.005 mol·kg^{-1}**											
**0.2**	997.63	996.33	994.83	992.84	990.78	**3**	998.91	997.80	996.35	994.70	992.89
**0.4**	997.65	996.35	994.85	992.86	990.79	**6**	998.92	997.81	996.36	994.72	992.90
**0.6**	997.66	996.38	994.86	992.87	990.80	**9**	998.93	997.83	996.42	994.79	992.94
**0.8**	997.67	996.41	994.88	992.89	990.81	**12**	999.02	997.89	996.48	994.84	993.00
**1.0**	997.68	996.43	994.91	992.90	990.81	**15**	999.10	997.96	996.53	994.89	993.05
**1.2**	997.69	996.46	994.93	992.91	990.81	**18**	999.19	998.01	996.58	994.94	993.10
**1.4**	997.71	996.47	994.95	992.92	990.82	**21**	999.25	998.06	996.62	994.98	993.13
**1.6**	997.72	996.48	994.97	992.92	990.82	**24**	999.31	998.10	996.67	995.02	993.17
**1.8**	997.73	996.49	994.99	992.93	990.82	**27**	999.35	998.14	996.71	995.07	993.20
**2.0**	997.74	996.50	995.00	992.94	990.83	**30**	999.40	998.18	996.74	995.10	993.23
**[Glycyl Dipeptide] = 0.010 mol·kg^{-1}**											
**0.2**	998.81	997.51	995.02	993.50	991.08	**3**	998.99	997.75	996.30	994.74	992.94
**0.4**	998.83	997.52	995.03	993.52	991.11	**6**	999.08	997.81	996.41	994.80	992.98
**0.6**	998.84	997.53	995.04	993.52	991.16	**9**	999.14	997.88	996.48	994.84	993.03
**0.8**	998.85	997.55	995.05	993.52	991.21	**12**	999.21	997.94	996.53	994.89	993.08
**1.0**	998.86	997.56	995.06	993.52	991.26	**15**	999.24	998.01	996.59	994.95	993.12
**1.2**	998.88	997.58	995.07	993.53	991.31	**18**	999.30	998.06	996.63	994.99	993.16
**1.4**	998.89	997.59	995.09	993.53	991.35	**21**	999.33	998.12	996.67	995.04	993.21
**1.6**	998.90	997.59	995.10	993.53	991.39	**24**	999.38	998.16	996.72	995.07	993.24
**1.8**	998.91	997.60	995.11	993.53	991.43	**27**	999.40	998.20	996.75	995.11	993.29
**2.0**	998.92	997.60	995.11	993.54	991.49	**30**	999.47	998.25	996.80	995.16	993.32

Table S2: Speed of sound, u ($m \cdot s^{-1}$) values for CTAB and DTAB in pure water and 0.001, 0.005 and 0.010 $mol \cdot kg^{-1}$aqueous solution of glycyl dipeptide at different temperatures.

CTAB						DTAB					
**[CTAB] mmol·kg⁻¹**	**293.15 K**	**298.15 K**	**303.15 K**	**308.15 K**	**313.15 K**	**[DTAB] mmol·kg⁻¹**	**293.15 K**	**298.15 K**	**303.15 K**	**308.15 K**	**313.15 K**
**[Pure water]**											
**0.2**	1482.40	1496.49	1509.18	1519.73	1528.84	**3**	1483.30	1497.20	1509.70	1520.30	1529.40
**0.4**	1482.54	1496.61	1509.26	1519.80	1528.91	**6**	1484.20	1497.90	1510.30	1520.90	1529.90
**0.6**	1482.70	1496.79	1509.33	1519.87	1528.99	**9**	1484.90	1498.60	1511.00	1521.50	1530.40
**0.8**	1482.81	1496.90	1509.43	1519.94	1529.07	**12**	1485.60	1499.20	1511.60	1522.00	1530.90
**1.0**	1482.99	1496.99	1509.47	1519.99	1529.13	**15**	1486.30	1499.90	1512.00	1522.60	1531.40
**1.2**	1483.07	1497.06	1509.51	1520.03	1529.16	**18**	1486.70	1500.20	1512.20	1522.60	1531.50
**1.4**	1483.15	1497.13	1509.55	1520.07	1529.20	**21**	1486.80	1500.30	1512.20	1522.60	1531.40
**1.6**	1483.23	1497.19	1509.59	1520.10	1529.23	**24**	1486.90	1500.30	1512.20	1522.60	1531.40
**1.8**	1483.29	1497.23	1509.62	1520.14	1529.26	**27**	1487.00	1500.40	1512.20	1522.60	1531.30
**2.0**	1483.38	1497.31	1509.66	1520.17	1529.30	**30**	1487.00	1500.40	1512.20	1522.50	1531.30
**[Glycyl Dipeptide] = 0.001 mol·kg⁻¹**											
**0.2**	1497.22	1509.52	1520.13	1528.14	1536.32	**3**	1484.45	1498.36	1510.52	1521.27	1529.96
**0.4**	1497.29	1509.64	1520.22	1528.19	1536.37	**6**	1484.93	1498.81	1511.01	1521.51	1530.34
**0.6**	1497.35	1509.73	1520.28	1528.22	1536.42	**9**	1485.65	1499.52	1511.62	1521.98	1530.94
**0.8**	1497.43	1509.79	1520.32	1528.24	1536.45	**12**	1486.47	1500.21	1512.12	1522.52	1531.45
**1.0**	1497.49	1509.89	1520.36	1528.27	1536.47	**15**	1487.06	1500.82	1512.65	1522.93	1531.89
**1.2**	1497.55	1509.96	1520.40	1528.29	1536.49	**18**	1487.36	1501.12	1512.92	1523.21	1532.15
**1.4**	1497.61	1509.99	1520.43	1528.31	1536.51	**21**	1487.58	1501.22	1513.07	1523.38	1532.42
**1.6**	1497.67	1510.05	1520.46	1528.33	1536.53	**24**	1487.69	1501.34	1513.11	1523.41	1532.54
**1.8**	1497.72	1510.11	1520.49	1528.35	1536.55	**27**	1487.86	1501.39	1513.15	1523.46	1532.59

**2.0**	1497.79	1510.16	1520.51	1528.37	1536.57	**30**	1487.98	1501.43	1513.20	1523.53	1532.61
**[Glycyl Dipeptide] = 0.005 mol·kg^{-1}**											
**0.2**	1498.06	1510.19	1521.43	1529.82	1537.36	**3**	1484.71	1498.57	1510.61	1521.41	1530.29
**0.4**	1498.16	1510.23	1521.52	1529.88	1537.44	**6**	1485.19	1499.05	1511.01	1521.68	1530.88
**0.6**	1498.22	1510.29	1521.63	1529.95	1537.49	**9**	1485.92	1499.76	1511.69	1522.32	1531.42
**0.8**	1498.32	1510.36	1521.67	1530.01	1537.56	**12**	1486.67	1500.43	1512.19	1522.89	1531.82
**1.0**	1498.44	1510.40	1521.73	1530.09	1537.61	**15**	1487.19	1500.91	1512.67	1523.39	1532.15
**1.2**	1498.51	1510.44	1521.79	1530.16	1537.68	**18**	1487.45	1501.16	1512.96	1523.59	1532.43
**1.4**	1498.62	1510.49	1521.86	1530.21	1537.75	**21**	1487.61	1501.27	1513.17	1523.63	1532.53
**1.6**	1498.51	1510.52	1521.92	1530.27	1537.81	**24**	1487.73	1501.39	1513.26	1523.68	1532.58
**1.8**	1498.62	1510.57	1521.96	1530.33	1537.87	**27**	1487.91	1501.45	1513.31	1523.72	1532.62
**2.0**	1498.71	1510.60	1521.96	1530.36	1537.96	**30**	1488.02	1501.52	1513.39	1523.77	1532.65
**[Glycyl Dipeptide] = 0.010 mol·kg^{-1}**											
**0.2**	1499.27	1511.13	1522.03	1531.75	1539.36	**3**	1484.99	1498.61	1510.69	1521.51	1530.33
**0.4**	1499.31	1511.17	1522.08	1531.80	1539.42	**6**	1485.32	1499.35	1511.31	1522.09	1530.95
**0.6**	1499.35	1511.22	1522.13	1531.85	1539.48	**9**	1486.03	1500.01	1511.92	1522.69	1531.51
**0.8**	1499.38	1511.26	1522.17	1531.89	1539.53	**12**	1486.83	1500.56	1512.55	1523.22	1532.06
**1.0**	1499.41	1511.29	1522.20	1531.92	1539.57	**15**	1487.28	1501.04	1512.99	1523.63	1532.36
**1.2**	1499.43	1511.31	1522.23	1531.94	1539.60	**18**	1487.54	1501.28	1513.18	1523.76	1532.61
**1.4**	1499.45	1511.33	1522.25	1531.96	1539.62	**21**	1487.68	1501.38	1513.29	1523.92	1532.68
**1.6**	1499.47	1511.34	1522.26	1531.98	1539.64	**24**	1487.76	1501.47	1513.36	1524.05	1532.72
**1.8**	1499.49	1511.35	1522.28	1532.00	1539.66	**27**	1487.95	1501.54	1513.43	1524.16	1532.78
**2.0**	1499.50	1511.36	1522.30	1532.01	1539.68	**30**	1488.09	1501.62	1513.49	1523.96	1532.77

Table S3: Viscosity, η (mPa·s) values for CTAB and DTAB in pure water and 0.001, 0.005 and 0.010 mol·kg^{-1} aqueous solution of glycyl dipeptide at different temperatures.

CTAB						DTAB					
**[CTAB] mmol·kg^{-1}**	**293.15 K**	**298.15 K**	**303.15 K**	**308.15 K**	**313.15 K**	**[DTAB] mmol·kg^{-1}**	**293.15 K**	**298.15 K**	**303.15 K**	**308.15 K**	**313.15 K**
**[Pure Water]**											
**0.2**	1.006	0.895	0.803	0.726	0.618	**3**	1.004	0.893	0.801	0.724	0.617
**0.4**	1.011	0.899	0.807	0.730	0.623	**6**	1.006	0.896	0.804	0.727	0.620
**0.6**	1.016	0.904	0.811	0.734	0.626	**9**	1.008	0.899	0.807	0.729	0.622
**0.8**	1.020	0.908	0.815	0.738	0.630	**12**	1.010	0.901	0.809	0.732	0.624
**1.0**	1.025	0.913	0.819	0.742	0.633	**15**	1.012	0.903	0.810	0.734	0.626
**1.2**	1.030	0.917	0.823	0.746	0.637	**18**	1.014	0.905	0.813	0.736	0.628
**1.4**	1.034	0.921	0.827	0.750	0.640	**21**	1.017	0.908	0.815	0.738	0.630
**1.6**	1.038	0.925	0.831	0.753	0.643	**24**	1.021	0.911	0.818	0.741	0.632
**1.8**	1.043	0.930	0.834	0.757	0.646	**27**	1.023	0.913	0.820	0.743	0.634
**2.0**	1.048	0.934	0.839	0.760	0.649	**30**	1.027	0.917	0.823	0.746	0.637
**[Glycyl Dipeptide] = 0.001 mol·kg^{-1}**											
**0.20**	1.425	1.271	1.130	1.076	0.923	**3**	1.181	1.090	1.063	1.044	0.967
**0.40**	1.445	1.286	1.145	1.093	0.936	**6**	1.188	1.097	1.068	1.047	0.971
**0.60**	1.460	1.300	1.165	1.110	0.953	**9**	1.197	1.128	1.073	1.054	0.975
**0.80**	1.476	1.319	1.182	1.125	0.967	**12**	1.204	1.111	1.079	1.060	0.979
**0.10**	1.495	1.339	1.200	1.145	0.985	**15**	1.211	1.118	1.082	1.064	0.984
**1.20**	1.510	1.350	1.220	1.162	1.001	**18**	1.217	1.126	1.087	1.070	0.989
**1.40**	1.524	1.363	1.239	1.177	1.016	**21**	1.227	1.134	1.093	1.072	0.995
**1.60**	1.536	1.379	1.257	1.194	1.029	**24**	1.232	1.141	1.096	1.076	0.997
**1.80**	1.547	1.400	1.272	1.212	1.042	**27**	1.239	1.146	1.101	1.078	1.002

**2.00**	1.564	1.420	1.289	1.233	1.061	**30**	1.245	1.154	1.105	1.080	1.004
**[Glycyl Dipeptide] = 0.005 mol·kg^{-1}**											
**0.20**	1.459	1.295	1.158	1.106	0.949	**3**	1.210	1.117	1.095	1.068	0.988
**0.40**	1.471	1.312	1.176	1.122	0.964	**6**	1.220	1.125	1.102	1.074	0.995
**0.60**	1.485	1.326	1.191	1.139	0.984	**9**	1.223	1.132	1.107	1.081	1.001
**0.80**	1.498	1.343	1.209	1.153	1.001	**12**	1.229	1.140	1.111	1.085	1.005
**0.10**	1.513	1.358	1.224	1.172	1.015	**15**	1.235	1.147	1.117	1.090	1.010
**1.20**	1.530	1.372	1.239	1.187	1.029	**18**	1.242	1.154	1.121	1.092	1.013
**1.40**	1.545	1.386	1.258	1.209	1.047	**21**	1.249	1.161	1.127	1.096	1.017
**1.60**	1.556	1.403	1.273	1.223	1.060	**24**	1.258	1.167	1.132	1.099	1.021
**1.80**	1.577	1.421	1.290	1.241	1.074	**27**	1.266	1.175	1.138	1.101	1.027
**2.00**	1.594	1.439	1.318	1.255	1.090	**30**	1.270	1.182	1.140	1.104	1.029
**[Glycyl Dipeptide] = 0.010 mol·kg^{-1}**											
**0.20**	1.474	1.317	1.182	1.131	0.979	**3**	1.241	1.165	1.148	1.080	1.005
**0.40**	1.488	1.334	1.206	1.145	0.992	**6**	1.256	1.175	1.156	1.089	1.013
**0.60**	1.508	1.346	1.225	1.162	1.005	**9**	1.270	1.183	1.164	1.096	1.019
**0.80**	1.530	1.363	1.246	1.176	1.022	**12**	1.280	1.191	1.171	1.102	1.025
**0.10**	1.543	1.381	1.274	1.193	1.035	**15**	1.288	1.196	1.178	1.108	1.031
**1.20**	1.559	1.398	1.294	1.211	1.042	**18**	1.302	1.205	1.185	1.116	1.037
**1.40**	1.575	1.416	1.312	1.224	1.056	**21**	1.306	1.213	1.191	1.120	1.041
**1.60**	1.593	1.433	1.332	1.238	1.069	**24**	1.310	1.217	1.198	1.126	1.047
**1.80**	1.610	1.447	1.353	1.252	1.082	**27**	1.314	1.220	1.205	1.132	1.051
**2.00**	1.624	1.459	1.371	1.266	1.096	**30**	1.321	1.225	1.212	1.135	1.057
